# Dopamine reverses reward insensitivity in apathy following globus pallidus lesions

**DOI:** 10.1016/j.cortex.2012.04.013

**Published:** 2013-05

**Authors:** Robert Adam, Alexander Leff, Nihal Sinha, Christopher Turner, Paul Bays, Bogdan Draganski, Masud Husain

**Affiliations:** a UCL Institute of Cognitive Neuroscience, London, UK; b UCL Institute of Neurology, London, UK; c LREN, Department des neurosciences cliniques - CHUV, Universite de Lausanne, Switzerland; d Max-Planck Institute for Human Cognitive and Brain Sciences

**Keywords:** Basal ganglia, Motivation, Effort, Stroke

## Abstract

Apathy is a complex, behavioural disorder associated with reduced spontaneous initiation of actions. Although present in mild forms in some healthy people, it is a pathological state in conditions such as Alzheimer's and Parkinson's disease where it can have profoundly devastating effects. Understanding the mechanisms underlying apathy is therefore of urgent concern but this has proven difficult because widespread brain changes in neurodegenerative diseases make interpretation difficult and there is no good animal model.

Here we present a very rare case with profound apathy following bilateral, focal lesions of the basal ganglia, with globus pallidus regions that connect with orbitofrontal (OFC) and ventromedial prefrontal cortex (VMPFC) particularly affected. Using two measures of oculomotor decision-making we show that apathy in this individual was associated with reward insensitivity. However, reward sensitivity could be established partially with levodopa and more effectively with a dopamine receptor agonist. Concomitantly, there was an improvement in the patient's clinical state, with reduced apathy, greater motivation and increased social interactions. These findings provide a model system to study a key neuropsychiatric disorder. They demonstrate that reward insensitivity associated with basal ganglia dysfunction might be an important component of apathy that can be reversed by dopaminergic modulation.

## 1 Introduction

Apathy is widespread in mild forms in many people. Recently it has become clear that it can be a severe behavioural condition in disorders such as Alzheimer's and Parkinson's disease (Marin, 1991; Starkstein and Leentjens, 2008). Defined as a state of impassivity associated with a lack of interest, concern or enthusiasm, apathy is dissociable from depression (Marin, 1991). But despite increasing awareness of the condition, we lack a good biological model. This is partly because attempts to understand underlying mechanisms in neurodegenerative diseases are difficult because of widespread brain changes. In addition it is now appreciated that apathy is unlikely to be a unitary construct but is more likely to be a syndrome that might result from dysfunction in several different component decision-making mechanisms (Levy and Dubois, 2006). Here, we investigate the possibility that one component of apathy might be relative insensitivity to rewards mediated by dysfunction in frontostriatal systems.

It has long been known that damage to medial frontal cortex can lead to an apathetic state, with patients demonstrating what has been termed ‘abulia’: reduced initiation of behaviour, lack of interest in their surroundings and loss of spontaneous emotional expression (Starkstein and Leentjens, 2008). A similar condition can also occur after focal lesions of the basal ganglia (Bhatia and Marsden, 1994), with the most severe presentations associated with bilateral damage (Laplane and Dubois, 2001; Schmidt et al., 2008). Such cases are relatively rare, however, and although many aspects of their behaviour have been reported, there has been very little experimental study (but see Schmidt et al., 2008).

Here we report one such individual with profound apathy following focal, bilateral lesions largely involving the globus pallidus (GPi) of the basal ganglia who provides a rare opportunity to understand both the neurobiology and pharmacological modulation of the condition. We used two oculomotor tasks designed to probe reward-based decision-making. In non-human primates, such behaviour has frequently been studied using eye movements, with internal globus pallidus (GPi) neurons demonstrating reward-related activity on such oculomotor tasks (Hong and Hikosaka, 2008; Shin and Sommer, 2010).

Although many brain regions, including parietal and temporal cortex, are activated by reward, a wide range of studies has now demonstrated that the basal ganglia, orbitofrontal cortex (OFC) and ventromedial prefrontal cortex (VMPFC) make a particularly important contribution to value-based decision-making (Haber and Knutson, 2010), with dopamine playing a critical role in modulating behavioural sensitivity to reward (Schultz, 2007). Emerging studies suggest that dopamine also makes a crucial contribution to effort-based decision-making, overcoming the cost of making efforts to obtain desired goals (Niv et al., 2007; Kurniawan et al., 2011).

Lesions of the medial frontal cortex affect how much effort rats are willing to invest for rewards (Walton et al., 2002, 2003; Rudebeck et al., 2006; Schweimer and Hauber, 2005). Rats are also rendered ‘anergic’ – employing less effortful feeding behaviour – by disruption of dopaminergic transmission in the nucleus accumbens (Font et al., 2008) or the GABA-ergic system in ventral pallidum (Farrar et al., 2008). Moreover, recent functional imaging in healthy humans implicates medial frontal and striatal regions in effort-based decision-making (Croxson et al., 2009). Taken together, these findings are consistent with the view that frontostriatal dysfunction might be a key component of apathy in human diseases (Cummings, 1993; Levy and Dubois, 2006), specifically by rendering patients unwilling to make efforts for rewards. They also point to the possibility that apathy might be amenable to modulation by dopamine, an hypothesis we were able to test in our rare case with bilateral GPi lesions.

## 2 Materials and methods

### 2.1 Participants

**Fig. 1:**
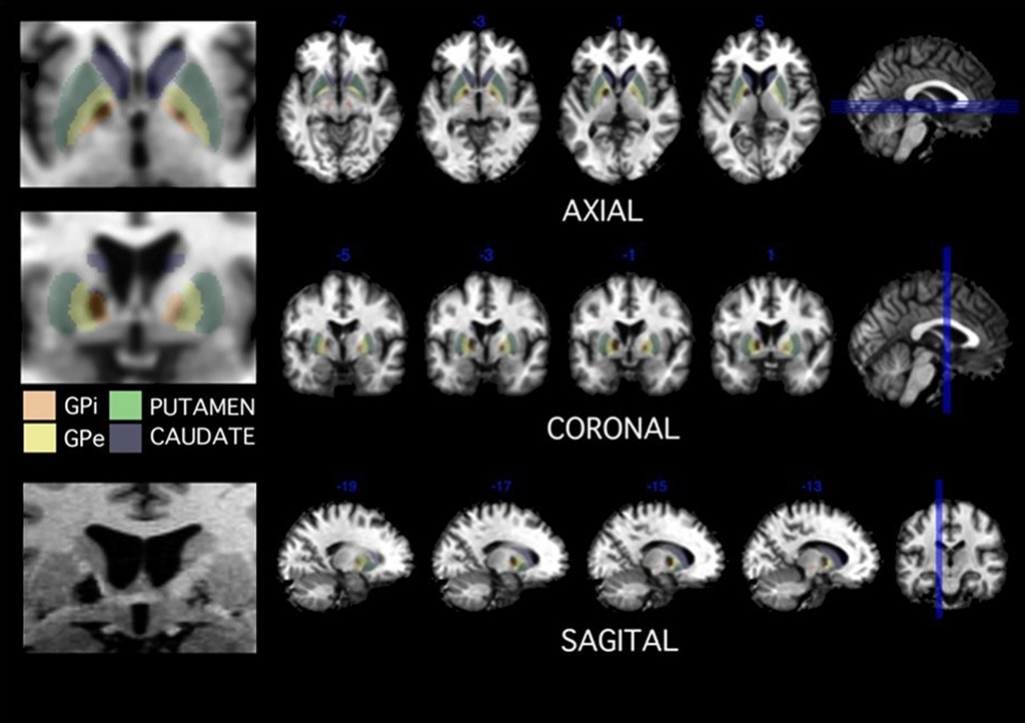
Sections demonstrating the extent of basal ganglia lesions. KD's GPi lesion was larger on the left than on the right. The lesions are projected onto boundaries of the GPi (orange), GPe (yellow), putamen (green) and caudate (purple). The bottom left coronal section is a close up at the level of the anterior commissure.

KD was a 41 year-old-male with ischaemic strokes affecting the internal segment of GPi bilaterally (Fig. 1), with greater involvement on the left. He recovered physically within days of his stroke but demonstrated reduced spontaneous and social activity. A previously exuberant and outgoing type, he became a reticent and reserved individual. He lacked interest in others and reduced spontaneity of action and thought. He remarked that his friends thought he had become boring. He was disinterested in going out to socialize.

He struggled or failed to achieve simple but important life goals such as returning to work. Indeed, he lost his job but then lacked the impetus even to seek unemployment benefit. After moving apartments, he failed to set up his music system because he “couldn't be bothered”, despite being an earnest enthusiast previously. He spent most of his day sitting at home, waiting for his flatmates to return and cook food.

Clinically, he was difficult to converse with. Questions were answered with short, closed responses. He did not initiate any lines of discussion, nor ask any questions. Although he was aware of his change in behaviour, he seemed to show little concern about his condition. He scored pathologically (8/12; scores >4 are abnormal) on the initiative and interest subscales of the Apathy Inventory (Robert et al., 2002). Despite demonstrating pronounced apathy, he did not complain of low mood nor seem objectively depressed. He denied biological symptoms of depression and did not score within the depressed range on several established scoring systems: 10 on Montgomery–Åsberg Depression Rating Scale (Montgomery and Åsberg, 1979), 7 on Beck Depression Inventory (Beck et al., 1988) and 2 on Hamilton rating scale for depression (Hamilton, 1960). Verbal and performance IQ were within the normal range.

Physical neurological examination, conducted independently by three consultant neurologists (authors AL, CT and MH) on four different occasions, consistently revealed normal tone, power and co-ordination in the limbs. There was no breakdown of fine finger movements or bradykinesia, even with distraction. Nor was there any evidence of dystonia or involuntary movement, such as chorea. Postural reflexes were intact and there was no abnormality of gait. Deep tendon reflexes and plantar responses were symmetrically normal. Saccadic, smooth pursuit and vergence eye movements were also unremarkable. Clinical single photon emission computed tomography (SPECT) revealed good presynaptic dopamine transporter (DAT) signal in the caudate and putamen, demonstrating integrity of the nigrostriatal dopaminergic pathway, consistent with lack of physical Parkinsonian signs. Because of the unusual nature of his strokes, a CT angiogram was performed but did not demonstrate any anomalous vasculature. Most such cases of bilateral basal ganglia infarction reported previously have no known established cause. The patient denied using 3,4-methylenedioxymethamphetamine (MDMA or “Ecstasy”), a substance which has very rarely been reported to be associated with basal ganglia infarction (Hanyu et al., 1995).

Healthy volunteers, [19 male, non-colour blind, mean age = 41 (SD 5.7); 12 right-handed] were recruited by website advertisement and from the UCL Psychology Department's subject pool, with local ethics committee approval. They completed both experimental tasks during a 1 h testing session. On the Barratt Impulsiveness Scale [BIS-11 (Patton et al., 1995)] their mean total score was 65.3 (SD 11.6). Written consent was obtained from all test subjects, according to the Declaration of Helsinki. The research studies reported here with KD started 9 months after his initial strokes.

### 2.2 Lesion anatomy and probabilistic diffusion tractography

**Fig. 2:**
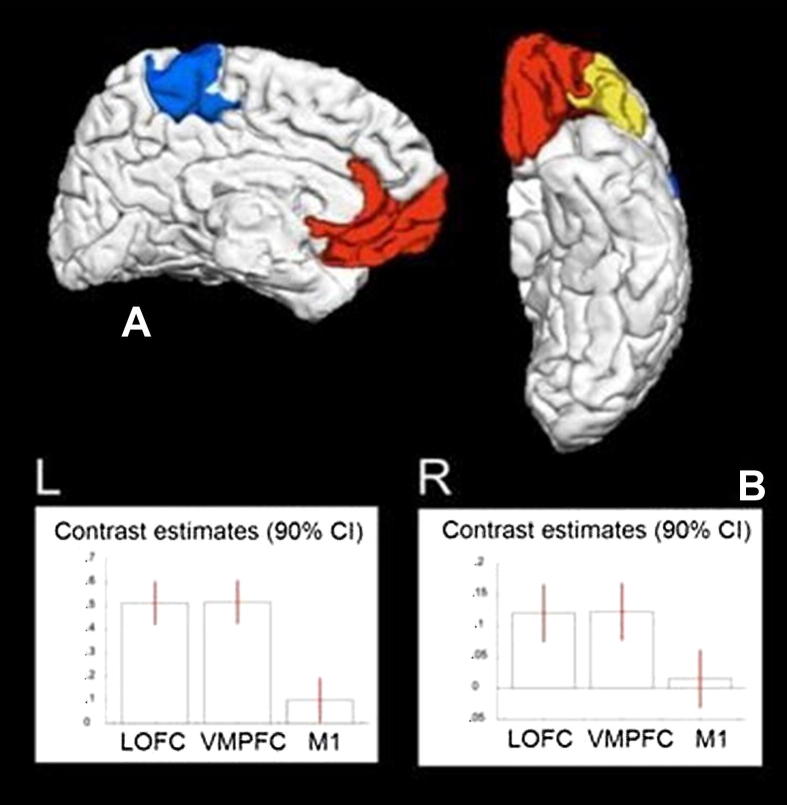
Lesions and cortical connections. (A) For DTI analysis, three cortical sites are shown: LOFC (yellow), VMPFC (red) and M1 (blue). (B) Regression coefficients (betas) extracted from the voxel of maximum intensity within the lesion on the left (L) and right (R) for the three tracts. High values indicate that the tract passes through the lesion with a high probability.

T1-weighted MR acquisitions of KD's brain were obtained at 1 × 1 × 1 mm resolution (Fig. 2A and B) on a 1.5 T Sonata Scanner (Siemens). Diffusion-weighted imaging (DWI) was performed with an echo planar sequence comprising a double spin-echo module to reduce the effect of eddy currents (Reese et al., 2003). Each data volume consisted of 40 axial slices of 2.3 mm thickness with no interslice gaps and an acquisition matrix of 96 × 96 in a field of view (FoV) of 220 × 220 mm, resulting in 2.3 mm^3^ isotropic voxels [echo time (TE), 90 msec; flip angle, 90°; fat saturation; bandwidth, 2003 Hz/pixel]. Each dataset consisted of 61 high-diffusion-weighted images (*b* = 1000 sec/mm^2^), with diffusion gradients applied along 61 evenly distributed diffusion directions obtained from a previously reported optimization procedure (Jansons and Alexander, 2003) and seven additional images with minimal diffusion weighting (*b* = 100 sec/mm^2^) and evenly distributed directions. The diffusion tensor was fitted using a standard linear least squares fit to the log measurements (Basser et al., 1994). Additionally, the fitting provides an effective *b* = 0 image. We also acquired high-resolution T1-weighted structural data using the modified driven equilibrium Fourier transform sequence [176 slices; 1 mm^3^ isotropic voxels; sagittal, phase encoding in anterior/posterior; FoV, 224 × 256 mm; matrix, 224 × 256; repetition time, 20.66 msec; TE, 8.42 msec; inversion time, 640 msec; flip angle, 25°; fat saturation; bandwidth, 178 Hz/pixel] (Deichmann, 2006).

Several recent human atlases were used to establish the extent of KD's lesions. Note that atrophy secondary to neuronal degeneration means that there is distortion of normal anatomy, in addition to the lesions themselves. It is therefore important to be familiar with such changes when interpreting these images. KD's lesions largely involved the GPi, more prominently on the left. There was no clear involvement of the habenula, subthalamic nucleus (STN), septum, medial hypothalamus, midline thalamic nuclei, and bed nucleus of stria terminalis, verified using a MR adapted version (Krauth et al., 2010) of the Morel histologically-based probabilistic atlas (Morel, 2007). Although part of the GPe may have been affected on the left, the lesions are largely within the GPi as shown in Fig. 1 of the text. Both the patient's MRI scan and the atlas were registered to the standardised Montreal Neurological Institute (MNI) space. We use a recently validated atlas of the pallidum (Prodoehl et al., 2008) and found lack of extensive involvement of the GPe.

In addition, to establish which cortical regions were most likely to be deafferented, diffusion-weighted data from 12 healthy aged-matched male subjects following the algorithm of Draganski et al. (2008). After automated cortical and subcortical parcellation using FreeSurfer (http://surfer.nmr.mgh.harvard.edu) we performed probabilistic diffusion tractography in subject-specific native space using a probabilistic index of connectivity (PICo) algorithm (Parker and Alexander, 2003, 2005) implemented in Camino software (http://www.cs.ucl.ac.uk/research/medic/camino/). To delineate the projection sites of specific cortical areas on the pallidum (Fig. 2A) we implemented a two stage probabilistic tractography approach: (i) probabilistic tractography from caudate to cortical targets as defined in FreeSurfer (LOFC – lateral orbitofrontal cortex, M1 – precentral and paracentral gyrus) and (ii) probabilistic tractography from pallidum to caudate after definition of the specific cortical projection sites. We calculated voxel-based PICo maps for the pallidum seed structures to each target area and transformed the individual maps to standard MNI space using parameter estimates from each individual's T1-weighted data.

Statistical analysis was performed within the SPM8 framework. After automated lesion detection using SPM8, we used KD's bipallidal lesion map in standard space to test the pattern of connectivity profiles of these lesion locations in 12 healthy subjects. The search volume was restricted to the internal and external pallidum as defined in the Basal Ganglia Human Area Template (Prodoehl et al., 2008). We tested the significance of the probability of the tracts passing through the lesion using an F-test: regression coefficients with 90% confidence intervals are presented in Fig. 2B. Post-hoc *t*-tests were used to identify differences in PICo between the three tracts to LOFC, VMPFC and M1. Data was thresholded at the level of *p* < .0001 uncorrected for multiple comparisons within the described search volume.

### 2.3 Experiment 1|traffic lights task (TLT)

**Fig. 3:**
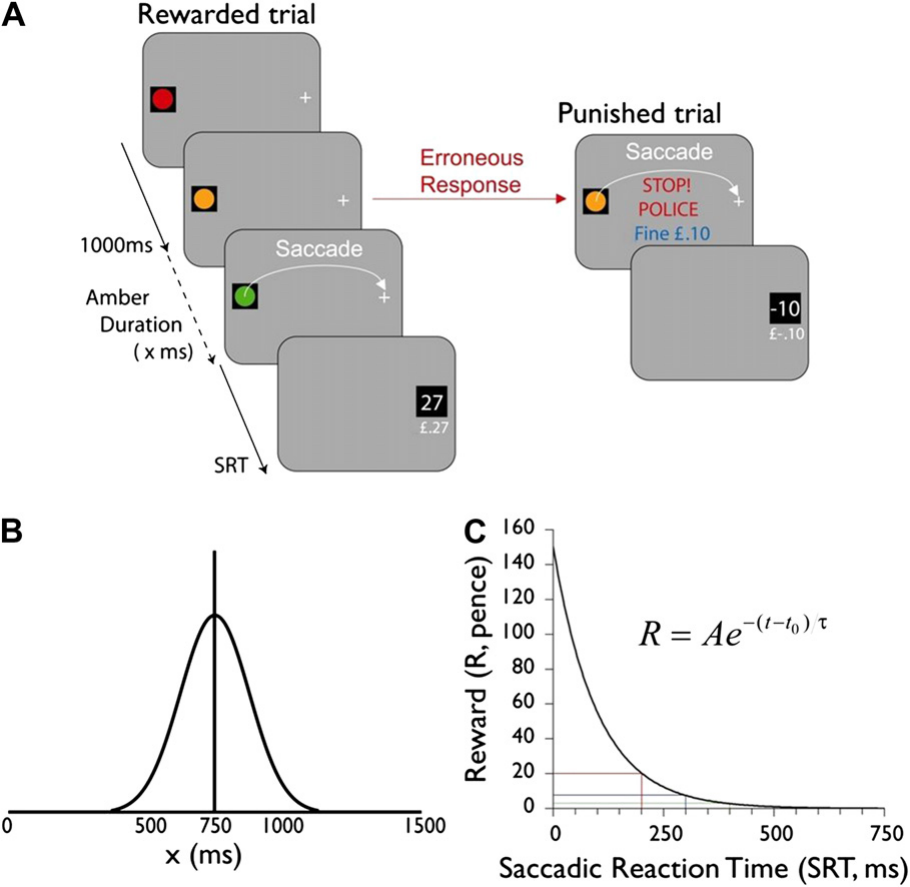
Traffic lights task (TLT). (A) Subjects fixated a circle which successively turned red, amber and green. They were required not to move their eyes until the onset of the green light, otherwise they receive a small (constant) fine or punishment. To maximize reward, participants had to make a saccade to the contralateral target as quickly as possible after green light onset. (B) Amber durations were selected at random from a normal distribution (mean = 750 msec, SD = 125 msec). (C) Reward was calculated with a hyperbolically decaying function with a maximum value of 150 pence (£1.50) when SRT was zero. Thus to maximize reward subjects should program an eye movement to coincide with green light onset. However, amber durations were not constant and therefore they either had to take a risk (high reward or punishment) or wait for the green light before programming a saccade (low reward).

We investigated rapid decision-making under risk for reward using a ‘traffic lights task’ (TLT) (Adam et al., 2012). Participants fixated a red light (3° diameter) for 1000 msec that successively turned amber and then green (Fig. 3) which was the signal to make a saccade to a target at 20° horizontal eccentricity. Amber duration was drawn probabilistically from a Gaussian distribution (mean 750 msec, SD 125 msec; Fig. 2B). Rewards depended upon saccadic reaction time (SRT), according to an exponential discounting function; Fig. 3C). Saccades made before green onset were penalized with a small, flat penalty.

Because saccades take ∼200 msec to initiate, any highly rewarded responses (latencies < 200 msec) have to be programmed before green onset. Thus to maximize outcome, subjects needed to make a decision about whether to initiate a response before the green light – and potentially obtain a high reward, but risk a penalty – or simply wait for the green light when they will receive a low reward. Participants were instructed to make as much money as possible. They performed ten blocks of fifty trials.

Reward (in pence) was calculated from acquiring the target using a decay function:

$$R=a\;e^{-\left(\frac{t-t_{0}}{k_{1}}\right)}$$

*a* = 150, *k*_1_ = 100 and *t* − *t*_0_ represents RT from green onset (msec).

Saccades made in advance of “GO!” were punished by a fixed fine of 10p. Rewards were displayed at the target site on each trial and a cumulative total was shown below this. Aural feedback was also given with a ‘ping’ for rewards of 0–19p, and a ‘ker-ching’ for rewards of 20p or more. An error trial was accompanied by a low pitched ‘beep’ in addition to a visual cue: “STOP Police! Fine £0.10”. Eye position was recorded using an EyeLink 1000 Hz eye tracker (SR Research Ltd, Ontario, Canada). Stimuli were displayed on a 22ʺ CRT monitor (150 Hz) at 60 cm.

#### 2.3.1 Linear rise-to-threshold modelling

It is not possible to establish definitively for any individual saccade whether it arose from an anticipatory or a reactive process. Because humans take ∼200 msec to plan and execute saccades, ‘reactive’ saccades – those made in response to green onset – are expected to have latencies of this order. Very early saccades (say < 50 msec after green onset) are likely to have been ‘anticipatory’, planned prior to green onset. However, there is a grey zone between these extremes.

We used an established method to decide how many of the saccades were s*tatistically most likely* to arise from each distribution, modelled by a linear rise-to-threshold process (Carpenter and Williams, 1995). We assumed two processes, one triggered by the amber light and the other by the green. Thus, the distribution of *reactive saccades* is described by a rapid rise-to-threshold process elicited by green onset. Whereas *anticipatory saccades* are described by a slower and independent rise-to-threshold process triggered by *amber* onset. A saccade is generated by whichever process reaches threshold first (Adam et al., 2012).

Maximum likelihood estimation provided best-fitting mean and variance parameters for each distribution. For controls, the model estimated a mean for the reactive distribution of 299 msec, SD 31 msec. We used a ‘cut off’ maximum saccadic RT of 200 msec, >3 SDs from this mean, to delineate *anticipatory* saccades.

### 2.4 Experiment 2|directional reward-sensitivity saccade task

**Fig. 4:**
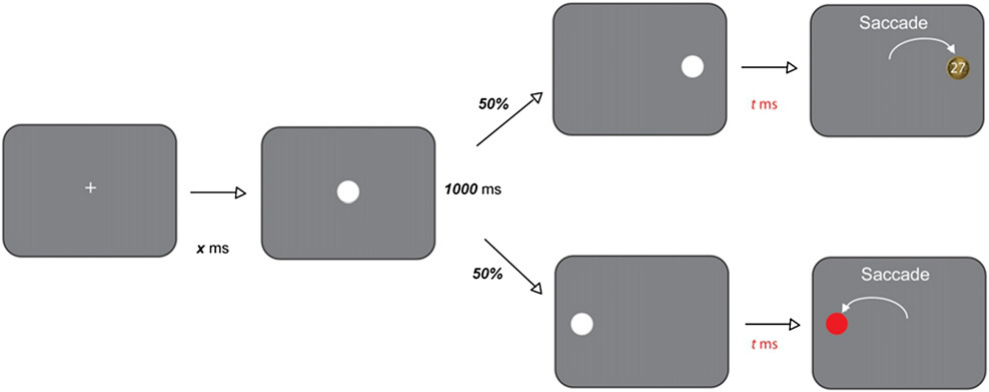
Directional saccadic reward task. Participants attended a central fixation spot which was extinguished after 1000 msec of fixation. They then made a saccade as fast as possible to a target presented either to the left or right (50% each side). One side was rewarded while the other received no reward. The rewarded side (RS) remained constant for an unpredictable number of trials before switching to the other side.

We also employed a second paradigm (Fig. 4) to investigate reward-dependent modulation of behaviour: speeding of saccades to rewarded targets (Hong and Hikosaka, 2008). Participants fixated a central cross (3° diameter) for 1000 msec and made saccades as quickly as possible to a target, 10° to the left or right (50% probability). Saccades to targets on only one side were rewarded depending upon reaction time (with a discounting function as for the TLT), and the rewarded side (RS) was altered, without warning, after a series of trials. Rewards were acknowledged by the display of a pound coin and a number representing the reward magnitude in pence. Reward value was dependent on latency using a function similar to that in the TLT. The RS changed every 10–14 trials. Participants performed two blocks of 120 trials. The difference in SRTs to the RS and unrewarded sides (US) was the measure of reward-sensitivity.

### 2.5 Dopaminergic drug challenges

KD received a single dose of Madopar 125 mg (100 mg l-dopa with a peripheral dopa-decarboxylase inhibitor, benserazide 25 mg), directly after the baseline tests. He was reassessed an hour later when peak l-dopa levels are reached. To assess whether any effects on l-dopa were due to simply more experience on the tasks, six controls were also tested an hour after performing their first session. A second group of controls (*N* = 12) also received the same dose of l-dopa but in double-blind randomized fashion, receiving placebo/drug one week apart.

KD was then given slowly increasing doses, reaching Madopar CR (long-acting preparation) 125 mg three times daily after eight weeks. Although there was moderate improvement in apathy, it was decided that there might be better response with a direct dopamine receptor agonist. l-dopa was therefore slowly discontinued and KD was off medication for 4 weeks (‘drug holiday’) before starting on the dopamine agonist ropinirole, initially .25 mg three times a day for 1 week, then increasing by .25 mg every week eventually to reach 1 mg thrice daily after three weeks. After a further four weeks he was established on 4 mg once daily of the long-acting formulation of ropinirole (Requip XL).

## 3 Results

### 3.1 Lesion anatomy and probabilistic tractography data

KD's lesions (Fig. 1) involved the GPi bilaterally, with greater involvement on the left. These lesions were not complete and it is important to note that part of the GPi was spared. Using a recently validated atlas of the pallidum (Prodoehl et al., 2008) we found only modest damage to GPe (external segment of the GPi) on the left. There was no involvement of the habenula, STN, septum, medial hypothalamus, midline thalamic nuclei, and bed nucleus of stria terminalis, verified using a MR adapted version (Krauth et al., 2010) of a histological atlas (Morel, 2007). Probabilistic diffusion tractography (Fig. 2) was used to examine the topography of pallidal connections to three cortical regions (Draganski et al., 2008). The region of GPi which is most strongly connected to LOFC and VMPFC was particularly affected, compared with projections to primary motor cortex (M1), more so on the left: VMFC > M1 left *Z* = 5.41, right *Z* = 3.51; LOFC > M1 *Z* = 5.33, right *Z* = 3.52 (all *p* < .001, uncorrected).

### 3.2 Experiments 1 and 2|baseline performance

**Fig. 5:**
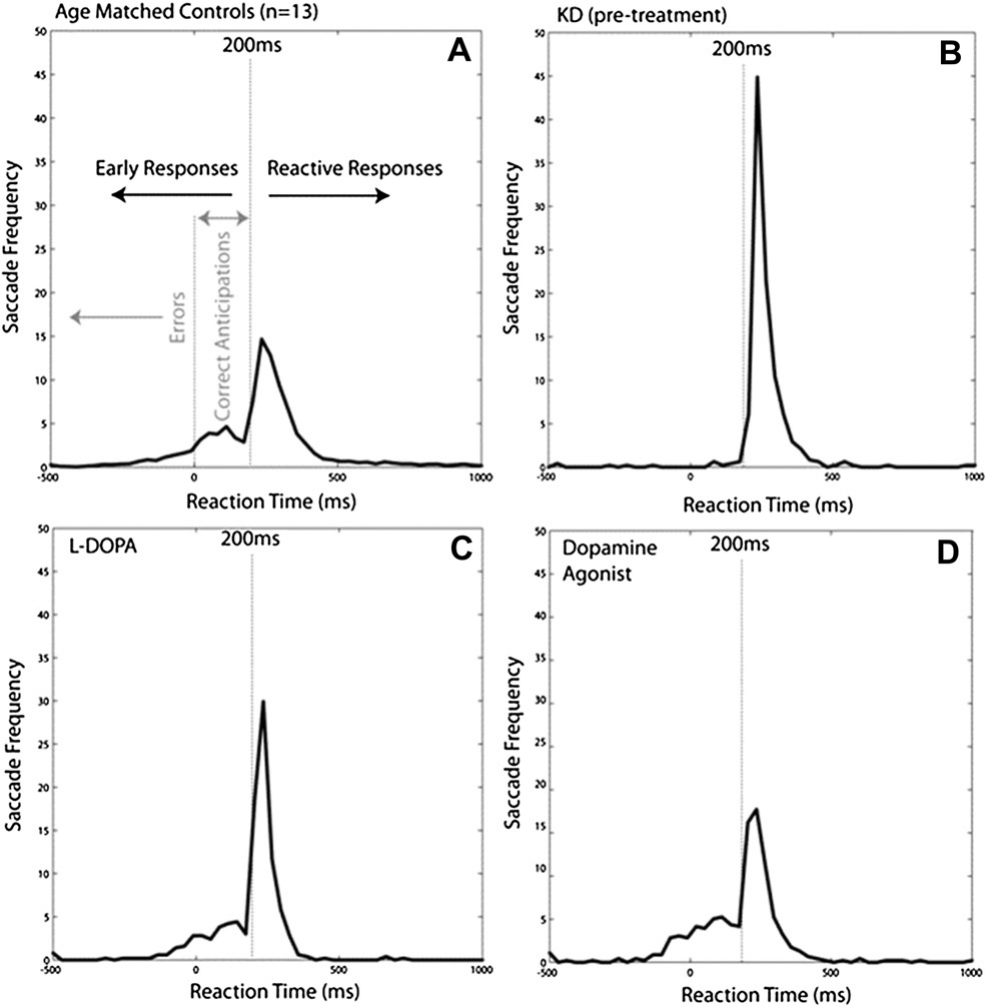
Traffic lights task (TLT): saccadic distributions. (A) Saccades for age-matched controls (*n* = 13) performing the TLT two distinct distributions: an early, anticipatory distribution and a later, reactive one made in response to green light onset. Early responses were divided into errors (saccades before the green light came on) and correct anticipations (saccades with < 200 msec latency after the green light). The plot here is for a total of 6500 saccades. (B) Pre-treatment, KD made mostly reactive saccades (461/500 trials [92.2%]) with a median latency of 248 msec. He made very few anticipatory saccades. (C) After treatment with l-DOPA 100 mg (Madopar CR 125 mg) three times a day for 12 weeks, there was a dramatic increase in early responding in KD. (D) After 12 weeks treatment with a dopamine agonist (ropinirole XL, 4 mg once a day), KD's distribution of saccades looks most similar to that of control subjects.

On the TLT (Fig. 3) SRTs in controls demonstrated a bimodal distribution (Fig. 5A). One population peaked ∼280 msec after green onset, consistent with saccades made ‘reactively’ following the GO signal. In addition, there was an early population with a peaking 63 msec after green onset. To demarcate these two distributions we used linear rise-to-threshold modelling, assuming two independent processes, the first triggered by amber light onset and the second by the green light (Adam et al., 2012). The early, anticipatory responses were further divided into errors (saccades before green onset) and correct anticipations (saccades after green onset, but planned in advance of it). ‘Reactive’ saccades were classified as those after 200 msec (see Methods).

Controls demonstrated a high proportion of early responses (mean 42% saccades, SD 18.95). Half were correct anticipations (21%, SD 8.64). The rest were errors (21%, SD 14.35). Overall mean Correct Anticipations: Errors Ratio (CA|ER) ratio was 1.53 (SD .87), with mean reward 18p/trial (SD 4.6p). CA|ER correlated well with mean reward obtained (*R*^2^ = .77; *p* < .0001).

In contrast, KD's distribution of saccades was unimodal, with most made after green onset (Fig. 5B). Nearly all his eye movements were reactive, with only 8.0% early responses, significantly different from controls (*Z* = 2.8, *p* = .003). Furthermore, the majority of these were errors; correct anticipations formed only 2.2% of saccades (*Z* = 2.8, *p* = .003). His CA|ER was .4 and he obtained only 14p/trial.

**Fig. 6:**
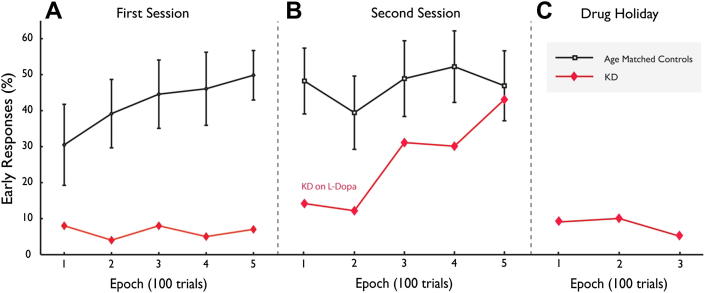
Percentage early responses on traffic lights task (TLT) over time. (A) Over the course of the first session, healthy controls showed increased early responses but KD did not. (B) In the second session, an hour later, controls showed no further change but KD 1 h after receiving l-dopa showed escalating early responses. (C) During the drug holiday period (off l-dopa), KD's early responses reverted to pre-treatment levels.

Within the first session, controls gradually increased the proportion of early responses (Fig. 6A), with a significant difference between the first 100 trials (30.5% early responses, SD 25.20) and the third (44.6%, 21.24; *p* < .05). There was also a trend for CA|ER to increase from the beginning to the end of the session (*p* = .08). In contrast to controls, KD showed no evidence of learning with 8% early responses in the first 100 trials to 7% in the last (Fig. 6A).

**Fig. 7:**
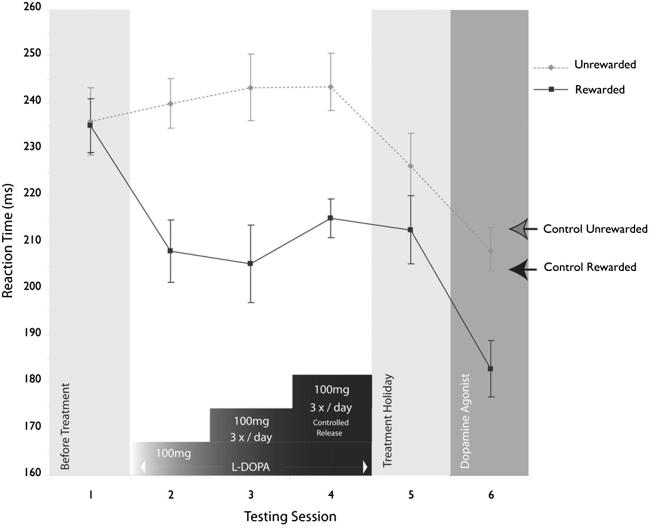
Results from the directional saccadic reward task. The control group (*n* = 12, arrows to side) showed a preference for the rewarded target locations, with significantly shorter SRTs. KD showed no reward preference at baseline, before treatment (Session 1). In Session 2 he was given a single dose (100 mg) of levodopa which led to a significant reward preference. This was maintained throughout chronic dopaminergic therapy (Sessions 3 Madopar 125 mg three times daily for 4 weeks, Session 4 Madopar CR 125 mg three times daily for 12 weeks). Following a treatment holiday (4 weeks), this reward preference was absent (Session 5). However, with subsequent treatment on the dopamine agonist ropinirole (1 mg three times a day), there was both a re-establishment of reward preference and significant decrease in latency to both rewarded and unrewarded targets. Error bars are +/− 1 SEM (standard error of the mean).

On the directional reward-sensitivity saccade task (Fig. 4) controls showed a small, but significant SRT advantage to the RS (mean RS 206 msec *vs* US 219 msec; *p* = .03) (Fig. 7). This sensitivity to reward did not change significantly over the first session [analysis of three forty-trial epochs *F*(5,66) = .24, *p* > .9]. By contrast, KD showed no significant difference between rewarded versus unrewarded saccades (mean US = 236 msec *vs* RS = 235 msec; *p* > .5; Fig. 7), and there was no significant change across epochs. His SRTs were longer than control means but within normal range.

### 3.3 Experiments 1 and 2|dopaminergic modulation

On the TLT, KD's performance altered dramatically 1 h after a single dose of l-dopa 100 mg (Figs. 5C and 6B). His early responses increased, with a CA|ER of 4.20 (6.67 SD > control mean of 2.20, SD .30) and overall increase in reward. Over the session, his early responses increased (14% in first 100 trials to 43% in the last; Fig. 6B).

Six controls also performed 500 trials an hour after the first session, but without l-dopa. Their proportion of early responses did not change significantly from the end of the first session (45%) to the end of the second (48%; *p* > .1; Fig. 6A and B). The same dose of l-dopa in 12 controls, tested in double-blind fashion, had no significant effect on SRTs (drug mean 306 msec, SD 121 *vs* 298 msec, SD 95 on placebo) or reward obtained (drug mean 23p/trial *vs* 24p/trial placebo). Thus l-dopa increased anticipatory saccades in KD but not in healthy people. The effect in KD was the largest increase in early responses from baseline of any subject who was tested twice, with or without l-dopa.

On the directional reward-sensitivity task (Fig. 7), following l-dopa KD now showed a markedly significant preference for the RS, apparent within the first epoch of forty trials (RS 211 msec *vs* US 238 msec; *p* = .002). Six subjects similarly performed a repeat session 1 h after the first, but without l-dopa. They demonstrated no further change in behaviour [*F*(11,60) = .7, *p* > .5]. In addition, eight controls tested in double-blind fashion on the same dose of l-dopa/placebo demonstrated reward-sensitivity, as previously. However, there was no further significant modulation by l-dopa (mean RS = 209 msec *vs* US = 219 msec placebo, *p* < .001; 214 msec and 219 msec on l-dopa, *p* < .01). Thus l-dopa speeded saccades to rewarded targets in KD but not in healthy people.

After eight weeks on l-dopa, KD showed moderate improvement in apathy. Concomitantly, the difference in SRT to US and RS was much larger than in controls, a consistent finding across all testing sessions (Fig. 7). Twelve weeks after initiating therapy, the difference between US and RS saccades was 36 msec (RS = 206 msec *vs* US = 242 msec; *p* < .0001). In isolation, these findings might be attributed to practice. However, SRTs to unrewarded targets actually increased while those to rewarded ones decreased, so the effects cannot be attributed to a simple generalized motor facilitation with practice and/or l-dopa.

On the TLT, performance reached a peak by 24 weeks l-dopa therapy when 33.4% of KD's saccades were now early responses, with 23.6% correct and 9.8% errors (CA|ER = 2.41 and mean reward now 23.2p/trial). However, a clinical decision was made to stop l-dopa and assess instead the effects of a dopamine agonist which acts directly at dopaminergic receptors.

Off medication, the difference in SRTs to RS and US targets became non-significant (Fig. 7), providing further evidence that reward-sensitivity observed in the previous sessions could not simply be attributed to practice. However, saccades were generally faster than before treatment, suggesting that there was some general practice effect that might have contributed non-specifically to speeding responses to both US and RS targets. On the TLT, off medication, the effects on l-dopa were also partly reversed with early responses strikingly reduced (Fig. 6C) and overall reward dipping to 13.7p/trial and CA|ER = .79.

KD started on an increasing dose of ropinirole, an agonist acting largely D2 and D3 dopamine receptors. By contrast, l-dopa would have a balanced effect across all these receptors by increasing synaptic dopamine. On 4 mg ropinirole daily there was marked improvement in KD's apathy. He was far more spontaneous in conversation, reported better social interactions and was more interested in events around him. He managed to secure a job and now scored in the normal range (4/12) on the initiative and interest subscales of the Apathy Inventory (Robert et al., 2002).

On the directional reward-sensitivity task, saccades were generally faster, but those to the RS were significantly faster (RS = 183 msec *vs* US = 208 msec; *p* < .001), far larger than in controls (Fig. 7). On the TLT by week four (on 4 mg ropinirole daily) KD demonstrated much greater early responding (45.2%). However, this was at the expense of greater numbers of errors (17.8% *vs* control mean = 24.2%) so the CA|ER (1.54) was not as high as on l-dopa. Despite this, mean reward (27.3p/trial) exceeded that achieved on l-dopa, matching the highest performing individual healthy control. Thus KD showing increased willingness to anticipate frequently and take risks, an effect that persisted over 12 weeks on ropinirole (Fig. 5D).

## 4 Discussion

We used novel probes of oculomotor decision-making to demonstrate relative insensitivity to reward in an individual with apathy following bilateral GPi lesions. Our TLT (Adam et al., 2012) requires reward sensitivity and motivation or effort to succeed, combined with fast reaction times and the ability to update behaviour in response to positive and negative feedback. A reactive response – simply waiting for the green light – is less well rewarded than an anticipatory response prepared in advance of the green signal. KD initially made very few anticipatory responses compared with age-matched controls. However, dopaminergic therapy, first with levodopa and then with ropinirole, increased anticipatory responses to within the normal range.

The directional saccade reward-sensitivity task, originally developed for the study of reward sensitivity in macaque monkeys (Hong and Hikosaka, 2008), demonstrated that KD had SRTs within the normal range but showed no speeding to the rewarded side (RS), unlike healthy volunteers. Treatment with levodopa led to reward sensitivity, with speeding of responses to the RS and slowing to the unrewarded side (US) compared to baseline. Off medication, the difference in SRTs to rewarded and unrewarded targets became non-significant, while subsequently on ropinirole, a direct dopamine D2/D3 receptor agonist, KD again demonstrated reward sensitivity, as well as generalized speeding.

These effects on dopaminergic medication were associated with clinical improvement – reduction of apathy and increased motivation to find work and in social interactions – most prominently while on the dopamine agonist. The findings demonstrate a causal relationship between basal ganglia function and motivation or willingness to make an effort for reward. They provide proof-of-concept data for the treatment of apathy which is increasingly recognized to be a key component of several neurological disorders (Bonelli and Cummings, 2008; Marin, 1991; Chow et al., 2009; Starkstein, 2009).

Unlike other tasks involving risk, such as the Iowa Gambling Task (Bechara et al., 1994) or the Cambridge Gamble Task (Clark et al., 2004), our TLT requires participants to take risks by making anticipatory responses. Many other paradigms place certain and risky options on an equal footing with the same amount of effort required for both choices. This has the benefit of establishing risk preferences independently of effort but tends to favour a careful, deliberative response strategy. The traffic lights paradigm imposes time constraints on decisions and rewards behaviour that might be considered ‘functionally impulsive’ (Dickman, 1990): on this task, it can be functionally useful to make anticipatory responses because these can lead to greater rewards, analogous to many situations in real life. It is possible that KD's lack of anticipatory responses on this task reflects *risk aversion*, rather than lack of motivation or unwillingness to make an effort for rewards. However, it is less easy to explain how such a mechanism might account for behaviour on the directional saccadic task, where there was no risk of incurring a penalty.

How did dopamine reverse apathy and reward insensitivity? Substantial evidence links dopamine to reinforcement learning (Schultz, 2007). However a growing body of research also implicates dopamine in effort-based decision-making, generating the motivation and vigour to overcome costs of initiating actions (Niv et al., 2007; Kurniawan et al., 2011). The progressive improvement of KD's performance on the TLT immediately post l-dopa (Fig. 6B) is suggestive of dopaminergic enhancement of learning. However, during the drug holiday period such learning was radically reversed (Fig. 6C), suggesting that if this effect was solely due to a reinforcement learning effect of l-dopa it had not been completely consolidated. Dopamine was still required to maintain it.

On the directional reward-sensitivity task, l-dopa also had a dramatic effect after its introduction, speeding saccades to the RS (Fig. 7). During the drug holiday, however, there was no longer any significant reward-sensitivity but saccades were generally faster than before treatment, suggesting there were some general, non-specific effects of practice on the task. The time course of action on reward-sensitivity and its reversal during the drug holiday makes it unlikely that dopaminergic effects on synaptic plasticity and learning were the only mechanism of action. Instead, it might also have had an effect on response vigour or overcoming costs of effort (Niv et al., 2007; Kurniawan et al., 2011).

Dopamine could act directly on brain systems left intact after stroke, but perhaps disconnected because the major outflow from the basal ganglia is via the GP. Alternatively, because the GPi lesions were not complete in KD, it is possible that his lesions led to imbalance in cross-talk between striatal regions which could be ameliorated by dopamine therapy. It has been demonstrated that parallel corticostriatal loops through the basal ganglia need not operate in isolation but can instead communicate with each other, e.g., via spiralling striato-nigro-striatal connections (Haber et al., 2000) which allow ventral striatal regions to influence more dorsal striatal areas. Moreover, the nigrostriatal system is not the only dopaminergic modulator of basal ganglia function; the intra-striatal dopaminergic system is complex and can alter with denervation (Smith and Kieval, 2000). Finally, it is important also to consider the possibility that the effects of dopamine observed in KD might arise from indirect, knock-on effects on other neurotransmitter systems, e.g., there is evidence of interactions between dopaminergic and noradrenergic systems (Hara et al., 2010) as well as several other neurotransmitters (see Steiner and Tseng, 2010, for reviews).

In macaques, using the directional reward saccade task, Hong and Hikosaka (2008) found that saccades to the RS with shorter latency than to the US, with reward-related speeding being associated with activity in GPi neurons which project to the lateral habenula. If a homologous circuit operates in the human brain, it is likely to have been partially disrupted in KD in whom both GPi were damaged. However, the lateral habenula remained intact, together with the caudate and putamen. Furthermore, SPECT imaging of the DAT demonstrated that the nigrostriatal dopaminergic pathway was intact as there was good signal bilaterally in the caudate and putamen of KD. Thus one locus of dopaminergic drug action is potentially the intact caudate, putamen or even surviving parts of the GP complex.

Another potential site of action of dopamine is prefrontal cortex. The OFC, in concert with basal ganglia structures, is considered to have a special role in the processing of reward signals (Schultz, 2000; Kringelbach and Rolls, 2004; Wallis, 2007). Projection of KD's lesion onto the known topography of the pallidal trans-thalamic connections to the cortex, determined using diffusion-weighted tractography (Draganski et al., 2008), suggests that the connections to the VMPFC and OFC have most likely been disrupted (Fig. 2). OFC neurons not only respond selectively to reward or aversive stimuli, but also signal relative preference for rewards and may integrate different types of information to compute a representation of value (Thorpe et al., 1983; Tremblay and Schultz, 1999; Padoa-Schioppa and Assad, 2006; Wallis and Kennerley, 2010). Consistent with these neurophysiological findings in macaque monkeys, imaging studies in humans have described activations in OFC and VMPFC which correlate with behavioural measures of stimulus value (O'Doherty, 2004; Plassmann et al., 2007; Rangel and Hare, 2010; Haber and Knutson, 2010; Glascher et al., 2009; Blair et al., 2006).

Lesions of the OFC in humans lead to impaired decision-making about the expected outcome of choices (Bechara et al., 1998) while alterations in striatal dopamine binding in drug addicts is associated with hypoactivity in OFC (Volkow et al., 2009). Dopaminergic neurons are known to innervate prefrontal cortex, including OFC (Williams and Goldman-Rakic, 1993). Although these arise from midbrain dopaminergic populations, partial disconnection of OFC neurons from trans-thalamic pallidal inputs – as is likely in KD – might disrupt dopaminergic reward signals within OFC. This view is compatible with recent functional imaging evidence that dopamine agonists might alter decision-making and risk-taking in susceptible individuals with Parkinson's disease via actions on OFC (van Eimeren et al., 2009).

Intriguingly, previous work also suggests that a dopaminergic deficit might be an important contributory factor to apathy in Parkinson's disease, which occurs in up to 60% of cases (Oguru et al., 2010). Patients who undergo STN deep brain stimulation (DBS) often require reduction or withdrawal of dopaminergic therapy because of improvements in motor control following surgery. Czernecki et al. (2008) reported that apathy occurred after dopamine withdrawal in some of these cases, but importantly it could be reversed with ropinirole. More recently, a PET study has demonstrated greater mesocorticolimbic dopaminergic denervation involving the OFC in Parkinson's disease patients who develop postoperative apathy compared to those who do not (Thobois et al., 2010).

Regardless of the precise locus of drug action in KD, it is clear that his lesions rendered him apathetic but this could be ameliorated by dopaminergic modulation. Alteration in reward-sensitivity mirrored clinical changes, suggesting apathy in this case is associated with lack of motivation to obtain rewards. Animal learning theory has proposed that rewards might in fact constitute the basic goals of voluntary behaviour (Dickinson and Balleine, 1994). From this perspective, the absence of sensitivity to rewards would be expected to have devastating consequences for goal-directed action, just as one observes in apathy. But note that although this view might account for behaviour in our particular case, apathy is most likely to be a syndrome that is multidimensional (Cummings, 1993; Levy and Dubois, 2006). In different clinical contexts, it could potentially result from deficits in other cognitive components of the decision-making process. Further studies are required to delineate these components and which specific deficits occur in different clinical conditions. Our study represents progress towards understanding one component of apathy – namely, relative reward insensitivity.

Although cases such as KD with bilateral GPi lesions are rare, apathy is common in Parkinson's disease (Oguru et al., 2010; Pedersen, et al., 2009; Starkstein, 2009), as well as in other neurodegenerative disorders, including Huntington's and Alzheimer's disease (Bonelli and Cummings, 2008; Chow et al., 2009; Starkstein et al., 2006; Marin, 1991). These conditions often involve disruption of cortico-striato-thalamo-cortical loops (Alexander et al., 1986) but the mechanisms underlying apathy when there is widespread neurodegeneration has been difficult to study. Focal lesion cases such as KD provide important information about the neural substrates underlying apathy and modulation of this behavioural state with neuropharmacological intervention.
